# Piloting a data dashboard to support data-informed health promotion in secondary schools: qualitative analysis of stakeholder interviews

**DOI:** 10.1186/s12889-026-26647-3

**Published:** 2026-02-18

**Authors:** J. Van Godwin, S. Long, B. Bowen, H. Reed, N. Page, M. Svobodova, M. Boffey, F. Rice, Y. Shenderovich, R. Bevan-Jones, S. Murphy, J. Segrott

**Affiliations:** 1 DECIPHer Centre, SPARK, Cardiff University, Cardiff, Wales UK; 2 Centre for Trials Research, School of Medicine, Cardiff University, Cardiff, Wales UK; 3 School Health Research Network, Cardiff University, Cardiff, Wales UK; 4 Wolfson Centre for Young People’s Mental Health, Division of Psychological Medicine and Clinical Neurosciences, Cardiff University, Cardiff, Wales UK; 5 Cwm Taf Morgannwg University Health Board, Pontypridd, Wales UK

**Keywords:** Health promotion, Schools, Student health and well-being, Data-informed practice, Data dashboard

## Abstract

**Background:**

Schools are called upon to use data-informed practice to support student health and well-being. However, they face implementation challenges including data accessibility and literacy. Data dashboards offer a method to address these challenges, thereby promoting data-informed practice. This paper reports on a pilot of The School Health Research Network Data Dashboard with secondary schools.

**Methods:**

The Dashboard was piloted by three secondary schools (recruited by size; free school meal entitlement) in Wales, UK. Interviews with school staff (*N* = 7) and public health practitioners (*N* = 6) were conducted. Data were analysed thematically. Research design, including interview questions, analytical interpretation and code, and theme development utilised Complex Adaptive Systems as the conceptual framework.

**Results:**

School staff had access to multiple data sets for health promotion but often lacked the time and capacity to utilise them. The Dashboard was perceived to be a user-friendly method of enhancing data accessibility and usability in secondary schools, providing appropriate training and guidance for school staff was available to avoid data misinterpretation. National roll-out of the Dashboard was supported if it aligned with the needs of schools and the wider education system.

**Conclusion:**

The Dashboard presents an opportunity for data-informed health and well-being practice and could also support schools to meet education system requirements, providing school staff receive appropriate training. This paper offers novel practical, policy-relevant insights on the interaction of digital dashboards with school systems, capacity constraints, and professional learning needs and valuable insights for public health systems seeking to support data-informed practice in educational settings.

**Supplementary Information:**

The online version contains supplementary material available at 10.1186/s12889-026-26647-3.

## Introduction

Promoting the health and well-being of children and young people is an international policy priority [1, 2] and schools are an important health promotion setting [3–5]. The World Health Organisation’s Health Promoting Schools framework advocates for a whole school approach to health promotion, incorporating the curriculum, schools’ physical and social environment and relationships with families [6, 7]. Developing Health Promoting Schools involves a range of stakeholders (staff, students, parents/carers, school governors, community partners) to promote health and well-being as an ongoing priority across different areas of schools’ work [8].

When developing strategies to promote child and adolescent health and well-being, schools are increasingly being called upon to adopt data-informed approaches [9–11]. Data gathered, for example, through self-report surveys, can support schools to identify need, monitor progress, and evaluate actions intended to promote health. Alongside school leadership, staff data literacy and capacity constraints play important roles in enabling or constraining data-informed practice within schools [10, 12, 13]. Further, research shows the resources required to collect, analyse and use data to inform school practices are not always readily available [11, 13].

The use of dashboards to promote data-informed practice has become an important strategy for research-practice infrastructures across a wide range of sectors, including national and regional population health [14], neighbourhood health [15], clinical supervision [16] and higher education [17]. An evaluation of a dashboard to support teacher data-driven decision-making found that it enhanced decision-making and data literacy, though the authors note that research on school-based dashboards is limited [18]. Data dashboards aim to enhance accessibility by summarising information in a way that can be understood quickly and easily, often employing visualisation to do so (e.g. graphics, charts). These features are designed to enable users to make effective and timely decisions about how best to use the data to inform ongoing practice (e.g. priority setting, selecting interventions to meet a need). Dashboards can also enable the visualisation of multiple dimensions of well-being data across various areas of health and well-being [19].

Data dashboards therefore have significant potential to address some of the key challenges to developing data-informed practice for health and well-being promotion in schools, including enhancing accessibility and usability. However, whilst there are examples of health promotion interventions which have employed aspects of school-level data collection [11], there is a paucity of research on the use of student health and well-being data dashboards. Little is therefore known about how schools use dashboards, make sense of the data being presented, or how data use informs health promotion actions. Such knowledge is critical to ensure that dashboards, implemented at scale, address schools’ needs. Questions also remain regarding the system-level support required for effective dashboard use (e.g. training) presenting a barrier to sustainable implementation.

The current study addresses this gap by exploring the acceptability, feasibility and training needs associated with rollout of a new digital school health and well-being data dashboard within the School Health Research Network (SHRN) in Wales, UK.

This paper also provides a unique contribution to understanding of data infrastructures for school health promotion literature by presenting findings from a pilot study exploring the implementation of a digital dashboard within school settings and its wider place within education and public health systems in Wales, UK. The Complex Adaptive Systems (CAS) framework is drawn upon to support this exploration, conceptualising schools as dynamic and interconnected organisations interacting with the Dashboard and wider systems which shape implementation and sustainability [10, 11, 20, 21]. Using qualitative interviews with school staff and regional school health and well-being public health practitioners, the study addressed the following research questions:

1. How do schools currently use data to support student health and well-being?
2. What are school staff and regional school health and well-being practitioners’ views on the acceptability and feasibility of using the SHRN data dashboard?
3. What professional learning and development (PLD) is required to enable school staff and regional school health and well-being practitioners to use the dashboard?
4. How should the dashboard be rolled out across Wales?

## Methods

### The SHRN data dashboard for schools

**Fig. 1:**
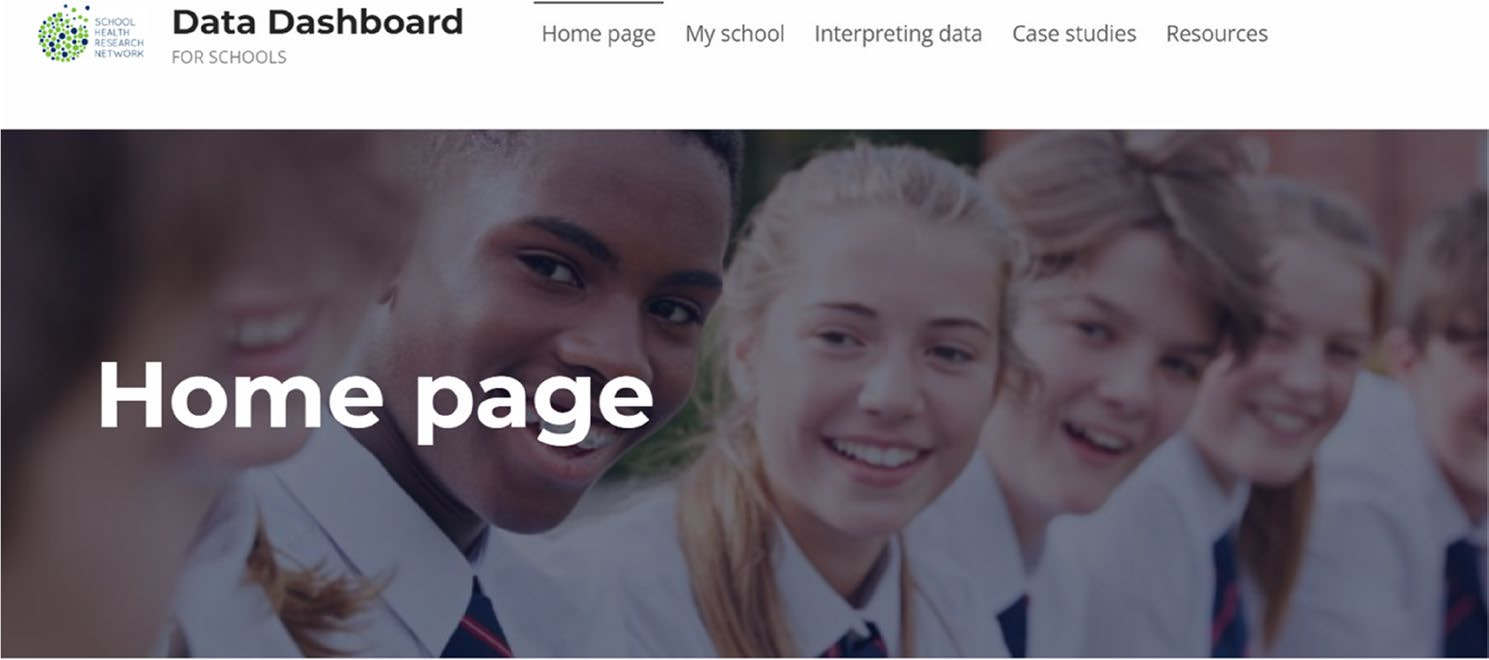
The Dashboard home page showing the five pages that can be navigated: ‘Home page’, ‘My school’, ‘Interpreting data’, ‘Case studies’, and ‘Resources’

**Fig. 2:**
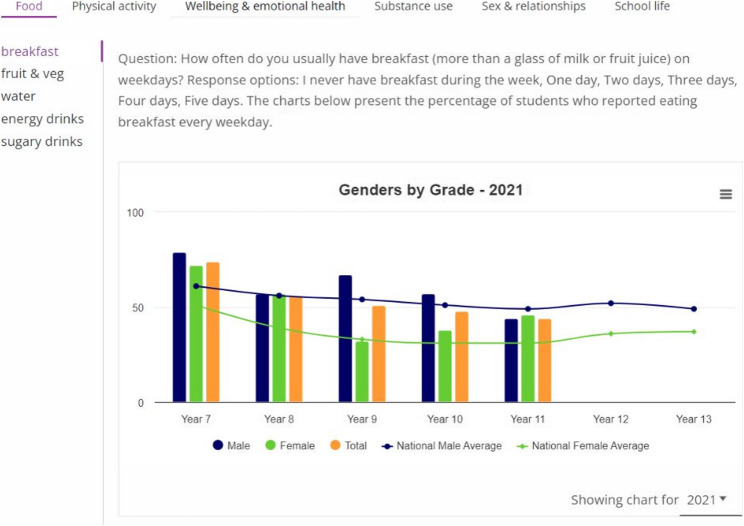
Schools’ aggregated data from the 2017, 2019 and 2021 survey waves categorised by theme (e.g. ‘food’) with topics (e.g. ‘breakfast’) displayed within each theme displayed vertically to the left of the charts

Established in 2013, SHRN is a strategic transdisciplinary partnership between education, health, academics and policy-makers, serving mainstream schools with students aged 11 to 18 years [21] and has been replicated in other countries and settings, including Scotland, English regions [11] and Namibia. Since 2017, over 90% of mainstream secondary schools in Wales have regularly participated in SHRN’s national health and well-being survey, with data collected from around 70% (approx. 130,000) of eligible secondary school-aged children each round [22]. Each participating school receives a tailored report containing aggregate data on key public health indicators (e.g. substance use, mental health and physical activity) to inform internal health promotion activities. The SHRN Data Dashboard For Schools (herein the Dashboard) was co-designed with school staff, young people and regional school health and well-being public health practitioners to support schools in using their research data from The SHRN Student Health and Well-being (SHW) survey [22]. It aims to improve data accessibility and understanding as well as promoting data use to inform health-promoting policies and action planning. A publication focused on the co-design and functionality of the Dashboard is currently in development. In short, the Dashboard provides bespoke, non-identifiable aggregate data covering a range of topics related to health and well-being (see Fig. 2) from student responses to the student completed SHRN SHW surveys in 2017, 2019 and 2021, including trends by age and gender. It contains five navigable sections (see Fig. 1, below) ‘Home’; ‘My school’; ‘Interpreting data’; ‘Case Studies’; and ‘Resources’. The ‘My School’ section displays aggregated data from the 2017, 2019 and 2021 survey waves, available to be categorised by themes such as ‘well-being and emotional health’ and ‘school life’ (see Fig. 2, below). Data tables can be downloaded. The ‘interpreting data’ section aims to help schools understand the charts while the ‘Case studies’ section gives examples of how some schools use SHRN data to address student well-being. The ‘resources’ section provides links to free resources to support data interpretation and use, such as Welsh Network of Health and Well-being Promoting Schools (WNHWPS) and Whole School Approach to Emotional and Mental Well-being: What Works Toolkit. National rollout of the Dashboard to every mainstream secondary is currently planned for 2026, though for this round, PDF reports will still be provided. However, moving beyond 2026, schools will receive their SHW data solely through the Dashboard. A prototype of the Dashboard is publicly available: https://mappedsites.cardiff.ac.uk/shrn-dash/.

### Sample and recruitment

Data collection involved two primary target populations - staff at secondary schools (pupils aged 11–18) and regional school health and well-being public health practitioners.

#### School recruitment

**Fig. 3:**
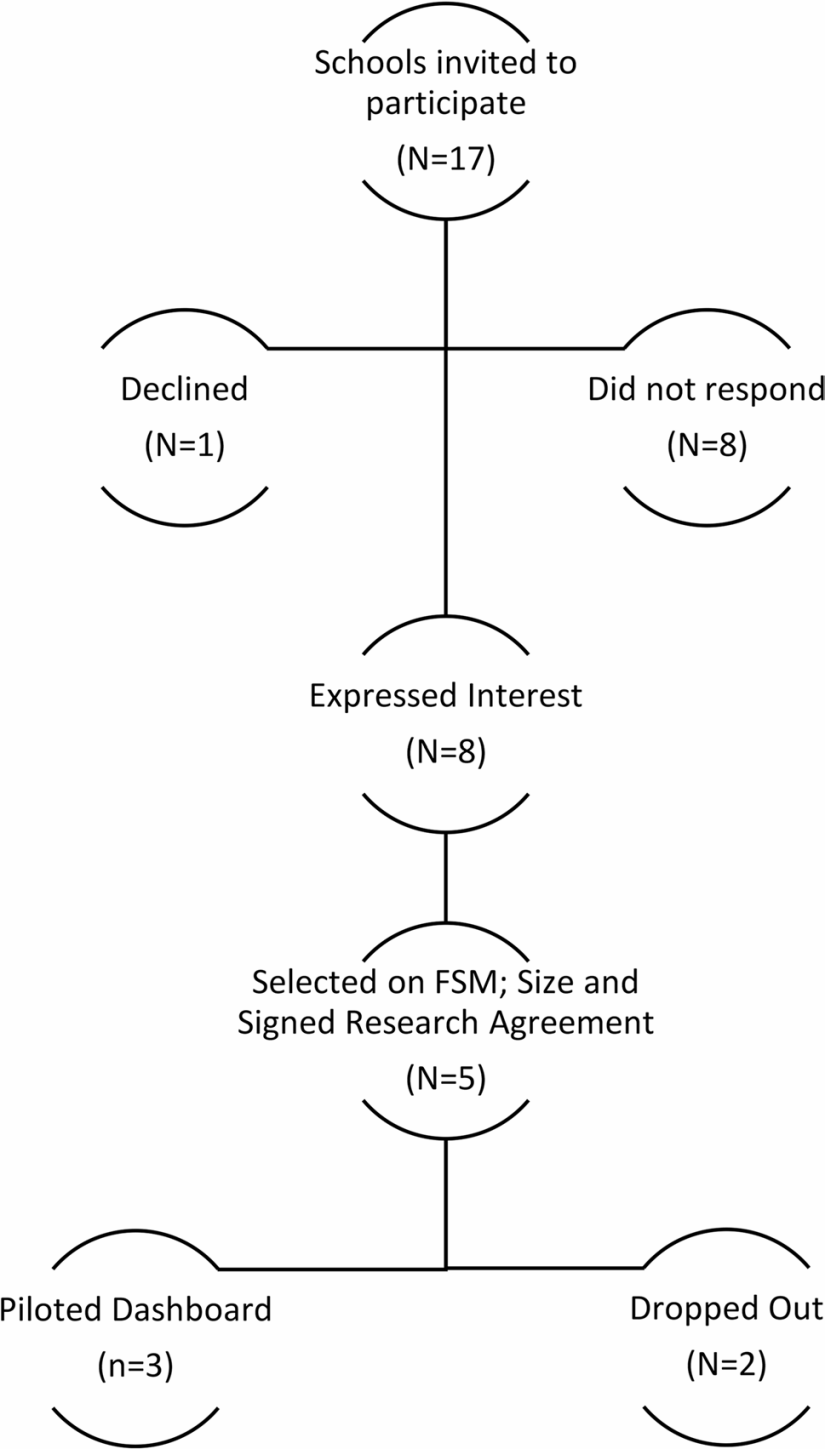
Secondary school sampling, recruitment and participation in piloting

Schools (*N* = 17) in South Wales were invited to participate in the pilot. Eight schools expressed interest in taking part, one declined to participate, and eight schools did not respond. Five secondary schools (students ages 11–18; mix of high and low free school meal entitlement [FSM] and school size) signed research agreements and were recruited to take part in the pilot. However, one school withdrew before data collection began. Figure 3, below, provides an overview of this process. Subsequently, School staff (*n* = 7) across four schools were asked to take part in three interviews spaced 2–6 weeks apart: (1) pre-Dashboard access; (2) 2–4 weeks post-Dashboard access; and (3) 8–10 weeks post-Dashboard access. As part of the pilot, schools were provided with a guidance document which provided a basic overview of Dashboard functionality, login details and a guide on how they could begin to use the Dashboard to support health promotion activities in their school (see supporting information 1). Three schools ultimately piloted the Dashboard as one school only took part in one interview and therefore did not pilot the Dashboard.

Schools were purposively sampled to allow for contextual diversity (see Table 1 below). Information on school size and FSM status in comparison to the national average was obtained from My Local School [23]. One school from each of the following four categories was initially selected: high FSM plus large school size; high FSM plus small school size; low FSM plus large school size; low FSM plus small school size, with an additional school selected due to the research team’s concern that a school may drop out. Decisions to include schools were made on a first come first serve basis, providing the schools represented the FSM and school size quota. Schools that expressed an interest were sent a research agreement setting out the terms of their participation in the study and the research team’s commitment to the schools. The three schools that took part in all three interviews and piloted the Dashboard received £500 once data collection was complete.

#### School staff interviews

**Fig. 4:**
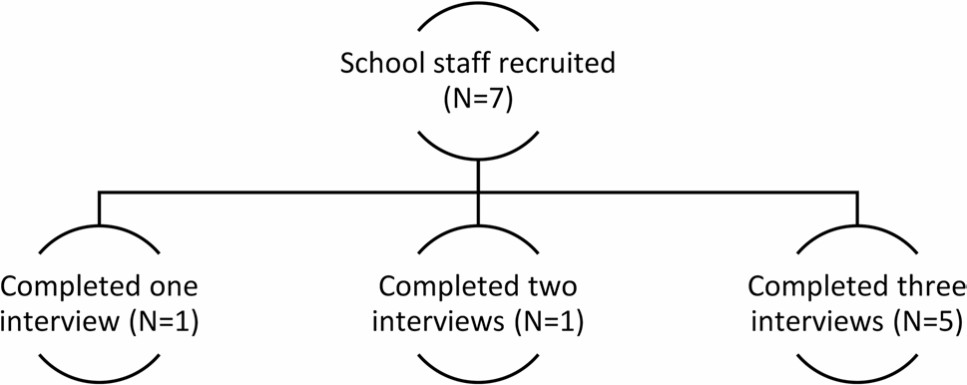
School staff recruitment and interview participation

School staff (*n* = 7) comprised members of the Senior Leadership Teams (SLT) and teaching staff with responsibility for student health and well-being. Staff (*n* = 5) from three of the four schools recruited took part in three rounds of interview, one member of staff from school four took part in two interviews and one member of staff from school two took part in one interview (see Fig. 4, below). All school staff signed electronic consent forms and interviews took place via Microsoft Teams.

**Table 1:** Overview of the four schools

Case Study School	Size	FSM	Staff Role and Interviews
School 1	Large [*N* = 1409]	Low [14%]	AHT *n* = 3 HWL *n* = 3
School 2*	Small [*N* = 863]	High [33%]	HWL *n* = 1
School 3	Large [*N* = 1813]	High [36%]	HWL *n* = 3 AOLE *n* = 3
School 4	Small [*N* = 692]	High [35%]	DHT *n* = 2 AHT *n* = 3

*Key*. *AHT* Assistant Head Teacher, *HWL* Health and Well-being Lead, *AOLE* Area of Learning Experience lead, *DHT* Deputy Head Teacher

^*^N.b. did not pilot dashboard

#### Regional school health and well-being public health practitioners

Health and Well-being Promoting Schools Co-ordinators (herein referred to as regional public health practitioners) are part of the Public Health Wales (PHW) Welsh Network of Health and Well-being Promoting Schools (WNHWPS) [24]. Each regional public health practitioner is linked regionally with several schools and provides support on all aspects of health promotion as per the World Health Organisation’s Health Promoting Schools Framework [24].

The study aimed to recruit regional public health practitioners linked to each of the four pilot schools through existing relationships. However, this did not prove possible. Instead, regional public health practitioners were recruited from across Wales to provide input regarding Dashboard acceptability and feasibility in the context of their roles and relationships with schools. Seven regional public health practitioners were approached via convenience sampling, of whom six consented to taking part in a one-off semi-structured interview. Regional public health practitioners interviewed represented areas in North, South, Mid, West and East Wales. No financial incentives were offered to regional public health practitioners for their participation in the interview. All regional public health practitioners signed electronic consent forms and interviews took place via Microsoft Teams.

### Ethics

This study was approved by the Research Ethics Committee, School of Social Sciences, Cardiff University in November 2023 (Ethics Reference Number: 509). All participants provided written informed consent to participate in this study. This study was conducted in accordance with the Declaration of Helsinki.

### Data collection

In total, 18 interviews with school staff (*n* = 7) were conducted across the four schools and six interviews with regional public health practitioners in February-May 2024. Interviews were transcribed verbatim and subjected to thematic analysis using NVivo 12 software. Aligned with the aims of the pilot, interviews explored current health and well-being data-informed practice in schools. Interviews then focused on the potential role of the Dashboard and the anticipated impact upon school health and well-being practice, including its acceptability, feasibility, staff training needs and future Dashboard rollout (additional file 1). Interviews lasted between 15 and 90 min. Interviewers (JVG and BB) attempted to ensure all questions in the topic guides were asked. For those not covered organically, interviewers prompted participants. The interviewers were able to build rapport with the participants and all interviews flowed smoothly, enabling questions in the schedule to be addressed and the exploration of other topics that emerged during the conversation. All interviews were audio-recorded, transcribed verbatim by a third-party transcription company, anonymised at the point of transcription, and checked for accuracy by JVG and BB.

### Data analysis

Thematic analysis [25, 26] was adopted to identify, analyse and report patterns within the data. Drawing on insights from a combination of initial interviews and study aims, the qualitative team (JVG, BB, SL) drafted initial codes. Two researchers (JVG, BB) then continued coding and met frequently to discuss areas of consensus and divergence, before the codes were refined. They then split and analysed the remaining transcripts generating further codes and themes. The qualitative team (JVG, BB, SL) met frequently together and with the wider MHDP team (JS; HR; NP; MS; MB) throughout the analysis process to seek alternative views of the data and discuss key thematic areas. Interview transcripts and subsequent codes and themes were reviewed and edited throughout data collection, analysis and write-up using Complex Adaptive Systems (CAS) as the conceptual framework.

A CAS perspective conceptualises schools as a set of interconnected components nested within wider education and country ‘supra’ systems [10, 11, 20, 21]. Within a complex system, individuals constantly interact with others to develop the work that they do (e.g. including daily routines, organisational norms, and new policies or interventions which they are asked to engage with). To shape analytical interpretation regarding schools data-informed practice and the adoption of the Dashboard, we drew on key tenets of a CAS approach. First, as complex systems, schools are not static systems, they are adaptive, constantly reacting to changes in the wider system. Second, systems have a tendency to self-organise [21], and will seek to make sense of, and bring order to new technologies or initiatives. Third, systems are sustained by feedback loops, with feedback on the impacts of a new way of working shaping subsequent actions that follow [21]. Adopting CAS as the conceptual framework informed the development and design of the interview guides and questions, analytical interpretation including code and theme development and revision. This included the use of CAS concepts such as informal and formal practice, feedback loops, open and closed system boundaries to inform the interpretation of the internal working of schools (e.g. between staff groups) and externally with key partners in education and supra systems. It also facilitated a detailed exploration of school practice that was formalised and structured within the system(s), practice that occurred informally, being led by individual(s) or that which occurred as part of the self-organisation of the system in response to external stimuli. New interventions, in this case the Dashboard, disrupt systems [27]. The CAS framework was therefore drawn upon to explore how and why schools interacted with the Dashboard in the ways they did, and the potential implications for its future implementation across different school and supra systems.

## Results

Three overarching themes were identified by the research team and framed through a CAS lens: (1) Formal and informal data-informed practice; and (2) Interaction with and perceived impact of the Dashboard; and (3) Sustainability and the wider education system. For themes two and three, a series of sub-themes were also identified (see Table 2 for an overview).

**Table 2:** Themes and sub-themes overview

Theme	Sub-theme(s)
1. Formal and informal data-informed practice	
2. Interaction with and perceived impact of the Dashboard	a. Initial familiarisation with the Dashboard b. Accessibility and functionality c. Meeting school staff needs
3. Sustainability and the wider education system	a. Dashboard rollout: Doing what’s right for schools b. The importance of alignment

Illustrative quotes are used and accompanied by anonymised participant IDs. School staff IDs use the following format, School ID (S1, S2 etc.); staff role (HWL=Health and Well-being Lead; SLT=Senior Leadership Team etc.); interview stage (01, 02 or 03). Regional public health practitioners IDs use the following format, HWPSC; numerical order in which the one-off interview took place (01, 02, 03 etc.).

### Formal and informal data-informed health and well-being practice

Interviews provided insight into the existing system dynamics related to school-based health and well-being practice. School staff and regional public health practitioners outlined how schools drew on multiple sources of data to understand student health and well-being and inform subsequent action planning. Schools recognised that data use comprised of many forms and should be integrated as part of a holistic approach to health and well-being. Some information was collected in a planned and systematic way, including routine data on attendance and attainment, information on vulnerable populations (e.g. safeguarding) and free school meal entitlement rates. More informal data was characterised by unplanned, ad-hoc conversations or observations by staff in the course of their daily work. Teacher observations, learning coaches, well-being officers, and external partners such as local youth teams also provided information through structured school processes, namely team meetings, and informally based on emergent school or student needs. Data insights were frequently informal instead of being part of a structured plan or strategy.

Data were also captured from student voice groups, assemblies and surveys with students. Schools discussed how information raised or shared ad-hoc by governors and parents and carers could be used to inform school-based health and well-being action. The dynamic nature of the school and wider community environment, with priorities emerging regarding health and wellbeing from these groups outlined how schools were responsive to wider emergent community issues and how these led to the self-organisation of practice and subsequent school-level action. Schools’ responsiveness to emergent factors, as well as formalised data collection mechanisms, demonstrated how they adapted and responded to need. Reflecting on the wealth of data they had access to, some school staff and regional public health practitioners perceived schools to be ‘data rich but time poor’:

“If anything, there’s too much data that schools now have, from attendance, attainment, whether it’s internal attainment tracking, external GCSE results. We then have the SHRN results. We then do a lot of student voice internally, which is quantifiable data in terms of strongly agreed, strongly disagreed, using the Estyn surveys. As well as kind of attitude to learning data from [actual] teachers, their academic progress data.” (S4DHT01).

The importance of SHRN student health and well-being data for influencing school practice was emphasised by school staff and regional public health practitioners and included informing curriculum content and cross-curricular activity, targeted school action and development plans, supporting policy initiatives, preparing for regulatory body school inspections, and targeted work with external partners. However, engagement with and use of SHRN data varied by school and individual staff member. A key limitation of SHRN data was its PDF format, with a teacher commenting that:

“they are long and […] unless somebody condenses it down, it’s not really accessible to all” (S1HWL01).

The same participant and some regional public health practitioners perceived that while SHRN was well established, some schools they had engaged with did not know how to utilise SHRN data and translate it into practice. This aligns with the wider challenges reported across school systems regarding the barriers facing school staff around the engagement, interpretation and use of student health and wellbeing data in health promotion practice.

### Interaction with and perceived impact of the Dashboard

#### Initial familiarisation with the Dashboard

Staff navigated the Dashboard in ways that aligned with both their needs and skills. It was apparent that interaction with other agents (in this case other staff members and students) was important. All three schools that piloted the Dashboard initially familiarised themselves with it prior to use. This process involved staff setting aside up to an hour, to log in and explore the Dashboard. Most staff reviewed the ‘My School’ section of the Dashboard which contained their school data from the biennial student health and well-being survey. The ‘Case Studies’ and ‘Interpretation of Data’ sections of the Dashboard, were not prioritised due to the limited time and capacity of staff. After initial familiarisation, staff in all three schools shared data insights informally through ad-hoc meetings, and formally using structures such as inset days, with colleagues in their SLT as well as wider staff in their school. A school also introduced the Dashboard to students in the classroom aiming to support curriculum delivery. A guide and checklist (see supplementary material 1) were provided by the research team to schools, these were only used to access Dashboard login details, however, staff preferred self-directed exploration. This indicates how, in light of capacity constraints, schools and staff adapt and self-organise in response to external requirements from wider systems and how these lead to emergent outcomes.

#### Accessibility and functionality

Participants were impressed with the presentation, colours and overall layout. Its accessibility in terms of ‘user friendliness’ - both in practice for schools piloting the Dashboard, and for regional public health practitioners in principle, was perceived as essential to its acceptability as a tool to support school development plans (SDPs), inform self-evaluation for the whole school approach to mental health and well-being and improve access to SHRN data and better aligned to responding to the dynamic, emergent and changing needs of schools. The user-friendliness was viewed as reducing the time it would take staff to access, interpret and ultimately utilise their SHRN data:

“I think the time element, whenever you try to do any sort of change, people say ’Oh, I haven’t got the time, haven’t got this’. Well, this is kind of solving that issue, isn’t it? Because it’s much easier to navigate, and you can just dip in and dip out and get exactly what you need.” (S1HWL02).

The user-friendliness of the Dashboard was also seen as an essential facilitator for staff less experienced or confident in their ability to use and interpret SHRN data. The Dashboard format meant that participants thought school data was more accessible compared to the current PDF and could therefore facilitate wider staff to engage with SHRN data:

“I think that [using the Dashboard] might be, rather than scrolling through a PDF, that would be a good way of doing it, a bit more interactive for them to use, isn’t it?…not just keeping it in SLT, using it in curriculum…there’s potentially more use in the curriculum having it electronically, isn’t there, and being able to manipulate a dashboard, and you look at it through there as a class from the students’ perspective as well.*”* (HWPSC05).

The Dashboard was viewed as providing the opportunity for continuous development, for example, by providing new case studies and resources:

“When you have a report, it’s just a report on paper. I know it’s an electronic copy, but you don’t have access to resources or case studies, or interpreting data, so they’re extra things, aren’t they, that’s beneficial? And if it’s going to be updated, if you’re going to update the case studies and put new ones in and maybe new resources coming in, it’s something that they can go back and check…” (HWPSC06).

Participants also valued how the Dashboard brought SHRN data from 2017, 2019 and 2021 into one place, allowing exploration of time trends - something that was more difficult to perform with the current PDF reports.

“…it’s easier to track trends over time, isn’t it? You can’t with a paper, you’d have to look at it. Because I had to do screenshots, because I used to get a paper copy, or […] e-copy, and I’d print it off. But I had to do screenshots then, for example, from the 2021-22 report to the 2019 report.…So that would be so far less time-consuming.” (HWPSC06).

Through access to time-trends, participants also described how the Dashboard enhanced the potential for measuring the impact of school programmes and interventions, both for schools and regional public health practitioners. This highlighted how user-friendliness and augmenting the process of staff accessing datasets can address the wider need across school systems for the simplification of engaging with and using data in practice.

The perceived improved layout and quicker access enabled increased sharing across staff could support staff to engage with the data more easily may lead to increased confidence in data use. This combined with a potential shift in perceptions of time barriers, with the sense that the data and information provided is now more dynamic (new case studies, data over time etc.), could lead to the formation of positive and reinforcing feedback loops which support Dashboard implementation and sustainability.

Some participants thought schools should also have the option of receiving their data in the current PDF format and described aspects of the PDFs which they valued and did not want to lose. Participants expressed concerns about how the change from PDF reports would affect the supporting resources and information for each SHRN topic and question:

“Obviously, the report as a PDF has kind of information regarding that particular topic area, doesn’t it, included in there, which is quite straightforward to look at?…It’s just making sure they access the right links to actually look at that information…” (HWPSC05).

Some school staff and regional public health practitioners felt that, for some schools, this would have the potential to negatively impact schools’ use of SHRN data. This demonstrates the risks associated with introducing wide-scale system change and the potential for this change to be ‘washed-out’ as it challenges established school practice.

Participants also mentioned that the Dashboard’s accessibility might vary based on data users’ literacy, health and well-being knowledge and skillset, including their technical expertise and confidence. Some considered a more open access approach for all school staff, whereas others were hesitant to share information due to concerns regarding data misinterpretation. Here the Dashboard is disrupting the system and instigating a change in practice, in response, school level work would likely vary depending on the existing school level dynamics and working relationships between staff.

Some regional public health practitioners highlighted the risk of schools misinterpreting the data and associating a causal relationship in school-level data with change where this may not be accurate.

“I hear schools saying a lot, which if you did put this into a Dashboard, is they talk about trends. And they say ‘Oh, you know, we’ve picked up a bit of a trend here’, and it’s like over two survey rounds. So, you know, sometimes they… they leap to conclusions about, you know, maybe two reports that they’re seeing… and if you’re going to move from a static PDF to putting them into years, the more that go on there, the more they’re going to think they’ve got, you know…there’s a lot more to identify in a trend than just a pattern over a couple of survey rounds.” (HWPSC01).

The need to ensure there was clarity at a school level on data access and sharing rights was also emphasised. Even though an established risk within current school practice rather than specific to the Dashboard, issues with IT and internet connections were considered a potential challenge for Dashboard use with one school piloting the Dashboard experiencing internet connection issues during the pilot. It was noted that this was not an issue with the current PDF reports. Moving forward it is essential that the Dashboard functions can be optimised to maintain system fit. Engagement with schools and staff during future evaluation of Dashboard implementation will be critical to ensuring the Dashboard’s relevance and alignment with ongoing and dynamic school and wider system changes.

#### Meeting school staff needs

Due to the Dashboard’s simple format, many school staff anticipated minimal training needs. However, according to regional public health practitioners, additional training and resources would be necessary, with a particular emphasis on understanding and utilising SHRN data. Some schools and regional public health practitioners suggested that once the SHRN or nominated in-school lead was trained on Dashboard use, it would be feasible and preferable for schools and staff to manage the introduction of the Dashboard internally as opposed to training being delivered by SHRN or another external provider. This would ensure its relevance to the individual school as well as enhance staff buy-in, emphasising the importance of interaction (not top down control) and collective action:

“[…] if there was training […] it would probably be for myself, and then I would disseminate that to those that require it. But, you know, in its layout at the moment, I think […] staff are used to looking at data. […] I think they would [be] able to navigate this. You know, it’d be absolutely fine.” (S4HWL02).

Opinions differed on the best format and structure for Dashboard training, suggested approaches included: in-person events, online; short cross-school events; and, having access to a training resource (either a document or video). For participants, the key factor was that any training and support offer accommodated staff need and availability. Some schools changed their perspective on what PLD may be needed after sharing the Dashboard with colleagues in their school. For these participants, before the Dashboard could be rolled out to their school staff SHRN specific training, an explanation of its measures, questions, data and overall purpose would be required:

“…it did make me sort of think I would need to kind of go through exactly what SHRN is, what the process [is] … you know? What you do with everybody so that everybody’s clear on sort of the context… the more I think about it, the more I think it needs to be sort of like quite in-depth training. So, I think in terms of if… if it is going to be more accessible to more staff, which [I] hope… you know? Which I want it to be, because I find it really useful, then I feel like I probably do need to invest a fair amount of time in training.” (S1HWL03).

This highlights the importance of piloting new approaches, in this case the Dashboard, with the target population and aligns with the wider recognised need for teachers to receive greater training and support for health and wellbeing practice in general and data-informed health and wellbeing practice, specifically.

A notable difference in perspectives between some school staff and regional public health practitioners was around the potential for misinterpretation of Dashboard data. Many school staff were confident in their individual ability - and that of colleagues, to engage with and understand the data presented on the Dashboards. However, others (and some regional public health practitioners) noted that, due to the pressured working environment, data literacy, limited time to engage with data and some staff not engaging fully with health and well-being tasks, the data on the Dashboard in its current format could be misinterpreted by staff who do not have experience or knowledge of data-informed practice. This risk appeared to be heightened given schools’ desire to share access to the Dashboard and the data more widely amongst staff compared to current SHRN data-sharing practice with data currently accessed by health and well-being leads and members of SLTs. It was suggested that the presentation of data should be reviewed and specifically the text to clarify what data represents should be added as not all staff may fully engage with the Dashboard, for example by only looking at data tables:

“But I think as an initial read of that, unless you read to the end of that summary, it’s maybe not as clear. I’m just thinking the people that maybe are in a rush, a bit busy, maybe might be like just easier to make something a bit more explicit in that sense.” (S3HWL03).

School staff also recommended that data tables from within and between topic areas should be able to be viewed side-by-side, for example comparing well-being to other areas of their data such as bullying, as well as view specific year groups’ progression over time to support the evaluation of impact and quality assurance processes of initiatives they had already undertaken.

The existing challenges schools face with increased expectation to engage in health promotion practice and gaps in health and well-being PLD for staff were also considered a potential challenge to Dashboard use as well as increasing the chance of data misinterpretation:

“There’s still, as far as I’m aware, no specific initial teacher training around health other than a bit of the curriculum and some safeguarding […] A lot of these people [school staff] are well-meaning and may have all sorts of skills. But […] it’s fair to assume that there will be gaps in their skills as well, because actually it’s just not trained in.” (HWPSC02).

Therefore, supporting staff data literacy during rollout was also viewed by some regional public health practitioners as key to address the risk of data being misinterpreted or misused. Teacher training is an essential aspect of the wider system shaping data use in schools and findings here indicate that this could address the historic gap in teacher training which has meant that both staff knowledge and practice regarding health and wellbeing is variable across school systems.

### Sustainability and the wider education system

#### Dashboard rollout: doing what’s right for schools

It was evident across the interviews that the interaction between schools and wider systems is crucial to both how the Dashboard is used and how it is rolled out. Participants generally supported national rollout of the Dashboard. Views were mixed on the next steps, including what support should be offered to schools. There was agreement that next steps should be informed by existing systemic pressures on the education system and expectations on school staff. Some participants suggested a need for established support pathways across national, regional and local systems. Others described a streamlined process of rollout and support for schools. Participants unanimously agreed that it was important to target the right time of year, avoiding periods that are particularly burdensome for schools such as in the build up to Christmas and during the spring/summer exams period. The suggested rollout timeframe of January 2026 was generally viewed positively by participants. The approach of rollout was not considered an issue for the schools piloting the Dashboard. However, they did note that some schools and staff may not be receptive to the change from PDF reports to the Dashboard, as some would prefer for all information on relevant resources and case studies to be collated in one place alongside the topic area. Participants including, both school staff and regional public health practitioners, also believed that some teachers would be resistant to change, preferring the current report style, format and method of data presentation.

Schools’ wider socio-political context was seen as key to the rollout of the Dashboard and school buy-in. Participants were conscious of the need to avoid putting schools in a position where they felt they were in competition with one another. It was felt that this traditional position of schools had been largely transcended in Wales, supported by the SHRN researcher-policy-practitioner partnership model and how it operated, namely ensuring schools have autonomy over who their data is shared with and avoiding judging schools’ performance based on SHRN data. There were concerns that the Dashboard could become a mechanism to comparatively judge school performance which would pose a risk to its implementation and sustainability. Regional public health practitioners were therefore keen to ensure schools retained the autonomy to decide who to their share data with, believing that current practice regarding how schools share their SHRN data with regional public health practitioners would largely remain similar. Once the Dashboard is in place, if schools decide to share their data with their local authority of public health teams, regional public health practitioners highlighted the importance ensuring the use of school data was conducted in the right way with decisions to allocate resources being data-informed based on the needs of students and schools. There was concern that if data was more widely shared this could lead to schools being ranked in certain areas of their work which would hurt the working relationship between schools and regional public health practitioners in the wider WNHWPS:

“We need to kind of keep that ethos in mind, that it’s the school’s data. We’ve moved away, and we’re hopefully moving away further from it in Wales, from the days where everything was a competition and a challenge, and schools [were] comparing themselves to one another, and yeah, this was more of a cooperative approach, so if you get the ethos right within the local authority, schools will share with me, but if we don’t get the ethos right, they won’t, and that’s external to that SHRN data approach itself.” (HWPSC03).

Participants also emphasised the importance of providing case studies for schools which demonstrated how to use the Dashboard in practice. This was seen as a method to promote engagement with the practical applications of the Dashboard as well as faster integration of data-informed practices to address existing expectations from the wider education system regarding the Curriculum for Wales (CfW) and the Whole School Approach to Mental Health and Well-being regarding health and well-being data collection and use. Case studies that accounted for contextual diversity were viewed as desirable. For example, schools with high FSM in areas of deprivation, wanted to see case studies from similar contexts. There was a view across participants, that too often case studies and good practice in general and through SHRN were based in schools whose context differed too greatly from their own to be relevant or applicable, thus impeding their ability to integrate the practice shared:

“…a bit of training about how it’s used, how like schools like us have used it within the teaching of the curriculum, how they use it with planning lessons, how they’re using it as a whole school approach, how we use it to support your self-evaluation” (S3HWL03).

#### The importance of alignment

Schools described several challenges which had the potential to impact the rollout, engagement with, and ultimate utilisation of the Dashboard. These included general workload burden, reduced resources, staffing and funding across school settings - which was anticipated to get worse over coming years:

“If you’re looking at what’s going on, they could all possibly boil down to capacity. Because at the moment, for example, you’ve got, you know, schools are still getting their heads around a new curriculum for Wales, there’s a lot in terms of cuts in education, so budgets are being cut, and, you know, they’re, losing 2% of their budgets.” (HWPSC01).

Participants agreed that it was essential therefore to align the Dashboard (and by default, SHRN) with requirements and expectations of schools from wider overlapping systems like the Whole School Approach to Mental Health and Well-being:

“maybe if you can get that sort of push from the local authority to say ‘Well, we do expect all schools to engage in this’, you know, ‘We want you all to be doing the survey’ ,and that’s why it might be useful to link in more with the whole school approach to the emotional and mental well-being framework as well. Do you know what I mean? So, it’s all just part of the expectation …” (S1AHT03).

In this case alignment was viewed in two ways, firstly that any data or information provided by schools regarding health and well-being would only need to be provided once rather than the process being duplicated in SHRN and the WSA. Secondly, that’s schools themselves would review the information they provide through SHRN to see how that could feed into the WSA or other requirements.

To enhance the alignment with existing requirements placed on schools and promote sustainability of use, some participants suggested that further functionality should be added to the Dashboard, allowing schools to download their raw data. This was viewed as allowing schools to integrate use of raw school data directly into their curriculum so students could be taught the process of analysing data, comparing their results with the SHRN results:

“So instead of just holding the chart, there will be an option for going back to the sample school so they can use the data for maths and science and that type of thing, so they could actually download the data in an Excel or just raw data output so that would be useful for a number of schools to actually reuse them.” (HWPSC03).

For some participants, there was hope that the introduction of the Dashboard could speed up the process of schools receiving their SHRN data to enable schools to take data-informed action sooner. Some stakeholders noted that the current delay between survey completion and data return could limit the timely use of SHRN data for school-level planning and action. Current time between schools completing SHRN surveys and receiving their data was seen as an existing limitation of the SHRN rather than a specific limitation of the Dashboard itself.

Both Dashboard rollout and alignment to the expectations and requirements placed on schools demonstrate the complexity of school systems and the supra systems within which they exist. The needs of schools and indeed the nature of the surrounding systems are dynamic and emergent, careful attention will need to be given to how the Dashboard interacts with them and the resulting changes or homeostasis.

## Discussion

This pilot study explored school staff and regional public health practitioners use of school health and well-being data to support action planning and health promotion activities within school systems. It also explored the acceptability, feasibility, staff training needs and rollout of the SHRN Data Dashboard for school health and well-being data. Schools were viewed as ‘data rich but time poor’, as participants noted that while schools had ample health and well-being data, the resources and capacity available for action planning were limited. The Dashboard was perceived to be an acceptable and feasible alternative to current SHRN data sharing processes. This was due to the perception that it could improve data-informed practices through user-friendliness, and augmentation of the process to access data. However, the provision of appropriate training was considered vital to avoid the perceived increased risk of misinterpretation or misuse of health and well-being data that could be an unintended consequence of enabling increased data access. Future roll-out of the Dashboard in secondary schools across Wales was supported providing it aligned to the wider needs of the education system. This related to both the context of Curriculum reform in Wales and the international movement towards a Whole School Approach to mental health and well-being, with this approach viewed as critical for the long-term sustainability of the Dashboard.

Great emphasis is placed on the importance of schools in promoting the health of children and young people [3–5] and schools are called upon to adopt data-informed approaches to promote child and adolescent health and well-being [9–11], . It is therefore essential to understand and address the factors that enable or constrain data use in schools [13]. Staff data literacy, capacity limitations and school leadership are all important factors [10, 12, 13]. As is the availability of the resource required to analyse and use data [11, 13]. Data dashboards have emerged as a potential means to address this and research has highlighted their importance in improving data-informed practices through user-friendliness and augmenting the process for accessing data for school staff [18]. Both of these factors were highlighted by participants when describing the SHRN Dashboard. The current paper, being one of very few previous studies having explored health and well-being-focused dashboards in school settings, provides some important insights here. Data systems and software, in this case the Dashboard, are simple and easy to use with datasets being gathered in one place. Subsequently this can reduce the time it takes to interpret and utilise data, therefore addressing schools’ need for a simplification of the processes involved in engaging with and utilising data.

While the Dashboard was positively received, some participants expressed a continued preference for the existing PDF format, citing its integrated presentation of data and supporting information. This format was perceived by some as more accessible and conducive for schools to share with external partners compared to the Dashboard, thereby facilitating collaboration with wider supra systems by supporting more open system boundaries. Future iterations of the Dashboard should address these concerns through the optimisation of Dashboard functions. Ongoing evaluation during the national implementation of the Dashboard will be critical to ensuring the Dashboard’s relevance, through meeting staff need, and long-term sustainability within the school setting.

The findings also build on previous research on school research networks [28] which identified that school staff prefer online hubs that act as a ‘one-stop-shop’ where data, case studies, wider evidence, and policy documents can all be stored, accessed and shared. Of particular importance was the provision of case studies and practical examples of who to engage with and how to use the Dashboard in multi-faceted ways was considered essential as it was a means to support staff with delivery in practice. This included using the Dashboard to support delivery of the CfW, informing school development plans (SDPs) and aligning with wider supra system requirements. More broadly, this can also be seen to be addressing the longstanding systemic challenge facing school staff to conduct data-informed practice, something expected in schools through both the CfW and WSA [29, 30]. Current research has also highlighted that sustainable school improvement initiatives involve elements of adaptability, capacity building, change and evolution [31]. The Dashboard addresses these factors as it is both a change and evolution of existing SHRN data sharing processes. According to participants it simplified the process of sharing with wider school staff and subsequently presented opportunities for capacity development. It also acted as a means to increase the use of SHRN data within the school setting. At present, SHRN data is not typically shared in a format that supports wide engagement across school teams and settings. The Dashboard, by offering what was perceived in this study to be a more accessible presentation of the data, has the potential to promote broader internal sharing and use. Although this format is new to schools, it was seen as a promising development for encouraging more generalised and cross-team engagement with SHRN data.

The challenges and potential unintended consequences of the Dashboard were also evident across interviews. The accessibility of the Dashboard and potential increase in the number of staff in each school accessing the Dashboard data was perceived by some to potentially lead to health and well-being work being based on a misinterpretation of the data. This highlights the importance of a PLD offer for schools in health and well-being data and evidence as a tool to mitigate the risk of the misinterpretation of data. This is something which has traditionally been absent from teacher training, therefore impacting the quality of staff health and well-being education and enhancing its variation across school settings [32–36]. The provision of guidance and support for school staff should be monitored through ongoing engagement with stakeholders in order to assess both its implementation and use.

There also seems to be a gap between different systems and stakeholders regarding school needs and what will and will not be possible with the Dashboard. Ethical and confidentiality issues, namely protecting the anonymity of individual students, mean that it would not be possible to share raw data with schools. However, this was something that some school staff would value and was seen to support the use and implementation of the Dashboard in curriculum activity. Notably, this desire did not factor in concerns about data literacy skills and the risk of data misinterpretation, which sharing raw data could exacerbate. Existing SHRN safeguards, which include the provision of non-identifiable aggregate data, disclosure control (estimates based on small numbers are suppressed to reduce volatility and increase robustness) and each school having a password protected, single user account can help mitigate against possible misinterpretation. However, it will remain an important consideration and the wider education and public health supra systems need to ensure that support structures continue to be or are, put in place to mitigate these risks.

There was also a hope that the introduction of the Dashboard would speed up the process of schools receiving their SHRN data once data collection was completed. However, this is a pre-existing challenge within the system and not specific to the Dashboard. It is currently not considered feasible to speed up the process of schools receiving their data, even when the Dashboard is introduced, as the national benchmarking provided in SHRN is only possible when all schools have completed the survey.

This research provides a unique contribution to the growing literature conceptualising schools as complex adaptive systems [10, 11, 20, 21, 31, 37], through the exploration of school-based health and well-being dashboards from a CAS perspective. The use of the CAS framework enabled the exploration of system requirements for data-informed practice, including the use of data dashboards and how they interact with schools, school staff and wider education and public health systems in relation to health promotion. Utilising a CAS lens, findings demonstrate the complexity of practice regarding data-informed practice which involved formal and informal elements that shaped decision-making and health and well-being practice. In this context, in response to resources and opportunities made available by the Dashboard, adaptation of the self-organisation within the system occurred. This included formation of collaborative initiatives by teachers with staff members not usually working with SHRN data and part of the schools’ health and well-being teams. Other forms of self-organisation included sharing data insights with colleagues *informally* through ad-hoc meetings and *formally* using structures such as inset days. An unanticipated application was also evident as some teachers reported having used the Dashboard in classroom settings to support curriculum delivery – something which was not initiated or guided by research team. The informal ways of working described in this paper, such as the use of informal data via unplanned, ad-hoc conversations or observations by staff in the course of their daily work, illustrate how schools ‘fill in gaps’ by creating their feedback loops to meet the needs of the increasing top-down pressure to use and act upon health and well-being data. It signals that schools are supported to create more formalised ways of working to promote data-driven practice, through networks like SHRN. The potential for the Dashboard to increase in-school collaboration could also enhance the integration of health and well-being data into day-to-day practice. Such integration is something which has been highlighted in the Welsh education systems as the ultimate aim [10].

The CAS lens also enabled exploration of the potential challenges with Dashboard introduction, roll out and sustainability. It is recommended that school improvement attempts should not adopt a one-size fits all model [38] and that a top-down standardised approach to address school improvement should be avoided [31]. However, the Dashboard will be scaled up and implemented across all secondary schools to support dissemination of bespoke data for health promotion. This decision has been informed by extensive engagement and co-design with school staff, students and external partners through the development of the Dashboard, something highlighted in the literature as essential to facilitate implementation and usability [19]. A detailed paper on this process is in development. The decision to develop and implement a data dashboard was also based on the recognition of schools’ needs regarding capacity limitations to engage with and use data to inform health and well-being practice alongside the desire to improve SHRN data-sharing processes. It is, however, possible that Dashboard rollout could still be viewed by some school staff as introducing a top-down change to current practice that is unexpected and potentially undesirable. Following the engagement and co-production model established in the development of the Dashboard, continued careful consideration needs to be given to the process for rolling out the Dashboard nationally. With sustained engagement with schools (staff and students) and regional practitioners’ (local authority and public health teams) needs essential. Simultaneously, any emerging unintended consequences or applications for schools’ data-informed practice should be explored. While the Dashboard was well received by all interview participants and has the potential to have a positive impact on school practice, the wider acceptability of the Dashboard will ultimately be driven by the perception of the Dashboard as a tool to either reduce or increase burden on schools and staff.

With limited availability of funding, high rates of socio-economic inequality and poor mental health among young people [39] the wider context facing schools in Wales is challenging. It is therefore vital to improve data and evidence engagement and use that aligns with wider supra systems including existing legislative, national and regional requirements and practices for schools. This can ensure these new ways of working actively reduce burden rather than exacerbating or merely displacing it. Since its inception, SHRN and the more recent WSA have served as collaborative areas for schools and regional public health practitioners. However, this research demonstrates the variation in collaborative practice across Wales at both the local level (between individual schools) and the regional level (between local authorities and health boards). Moving forward, the ongoing interaction of the Dashboard with these contexts and relationships requires attention, something which can be facilitated through a CAS approach.

Participants in this study highlighted the importance of ensuring digitalisation of school practice did not lead to increased measurement and expectation regarding effectiveness. Data use is only one aspect of schools’ approach to student health and well-being and should be considered as part of their holistic, WSA to health and well-being rather than being the sole driver of action.

### Implications for policy and practice

Through the adoption of a CAS lens, this research illustrates how data dashboards can support the long-term development of data-informed health promotion practices within schools. The ways in which dashboards interact with school systems is critical to understanding the likely success of their adoption and integration. How staff are supported and trained to understand and use data, and the extent to which dashboards minimise the burden placed on those tasked with navigating them, is key. More broadly, the use of research data to support the growing emphasis on health promotion in the education system will require alignment across a complex range of actors, including schools and young people, public health practitioners, national policy-makers, and academic researchers. The initial work in co-producing the Dashboard with key users in the system, and the current work in piloting its implementation are designed to maximise its value and sustainability. Conceptualising the Dashboard as an ‘event’ within complex social systems [27], there remains a risk of the Dashboard getting ‘washed out’ of school practice if it not integrated into routine practice. Integration into routine practice can be facilitated at a national level through policy initiatives such as CfW and WSA and at a regional and local level through Wales’ answer to WHO’s WSA initiative - the Welsh Network of Health and Well-being Promoting Schools. Wales is in a unique position for other countries to learn regarding its school health and well-being systems with the SHRN data dashboard forming part of these systems.

Future uses of the Dashboard have the potential to be multi-faceted. At the school level they include further integration of data-informed practice into the curriculum; use as a tool to increase data skills of school staff, as demonstrated in research on education dashboards [18]; and, enhancing the process of monitoring and measuring Health and Well-being interventions.

### Strengths, limitations and future work

Our study provides important insight regarding how data-informed health promotion within schools sits within the broader system, linking with regional public health practitioners, other schools across Wales, national policy directives (e.g. on a WSA to mental health) and the central resources provided by the SHRN team. The study took place within a small number of schools – and those which may have been more engaged in using SHRN data, therefore the findings related to the current view of and use of SHRN as well as the future use of SHRN and the Dashboard are not representative of schools outside of this sample. A potential limitation of the current study relates to attrition. Given one school withdrew prior to data collection and another following first round interviews, meaning they did not pilot the dashboard, it is possible that alternative or contrary views were not recorded. For both schools that withdrew, capacity was identified as the key barrier to participation. This likely reflects wider systemic issues, also captured in this study, which may prevent schools from adopting new approaches into their practice. Despite the small sample, by using CAS as a conceptual framework it has been possible to gather an understanding of potential strategies for the implementation and integration of the Dashboard (including barriers and facilitators) in schools across Wales has been generated. It is essential that this is monitored on an ongoing basis and more schools and staff are actively engaged with. Finally, in an attempt to limit bias, the pilot team who undertook data collection for the pilot were different to the team that ran the co-development process. As SHRN moves ahead with the national rollout of the Dashboard, there are plans to evaluate its implementation at scale and to further develop an understanding of how it interacts with school systems across different contexts.

## Conclusion

The SHRN Data Dashboard for Schools was considered to have a user-friendly design and perceived to enhance schools’ ability to conduct data-informed practice using SHRN data. It presents an opportunity for data-informed health and well-being practice, providing appropriate training and support around data use is offered. Digitising school health and well-being data has the potential to improve the accessibility and adoption of data-informed health and well-being promotion practice among schools therefore supporting schools to meet wider requirements of the education system. This paper addresses an important and under-researched area, namely, the use of digital infrastructures for school health promotion. Findings offer both practical and policy-relevant insights into how digital dashboards interact with school systems, including capacity constraints and professional learning needs. Therefore providing a unique contribution to research on schools as complex adaptive systems. Additionally, this research provides valuable insights for wider public health systems on how to support educational settings to undertake data-informed practice. Future research should explore the impact of Dashboard rollout on school, wider educational, and public health systems.

## Supplementary Information

Supplementary Material 1.

## Data Availability

Research data are not shared.

## Abbreviations

CAS: Complex Adaptive Systems
CfW: Curriculum for Wales
HPS: Health Promoting Schools
HWPSCs: Health and Well-being Promoting Schools Co-ordinators
PHW: Public Health Wales
PLD: Professional Learning Development
SDP: School Development Plan
SHRN: School Health Research Network
SHW: Student Health and Well-being Survey
SLT: Senior Leadership Team
WHO: World Health Organization
WNHWPS: Welsh Network of Health and Well-being Promoting Schools
WSA: Whole school approach
