# Supplementary material for: Piloting a data dashboard to support data-informed health promotion in secondary schools: qualitative analysis of stakeholder interviews
Source: BMC Public Health. 2026 Feb 18;26:987. doi: 10.1186/s12889-026-26647-3 (PMC13020240; doi:10.1186/s12889-026-26647-3)
Supplement: Supplementary file 1 — Supplementary Material 1. [file 12889_2026_26647_MOESM1_ESM.docx]

# Supporting Information 1

1. **Schools Checklist**

The checklist below should be completed by the lead for this work with input from the wider school team. The checklist will also be used to inform your follow-up interviews. Please ensure you have read the introductory guidance on how to access your Dashboard above and let the research team know if you have any problems in accessing the Dashboard.

| **Task** | **Notes and reflections (please answer the questions below)** |
| --- | --- |
| 1. Log in and familiarize yourself with the dashboard | What are your initial thoughts and reflections on the Dashboard? |
| 1. Bring together a team of 2-4 staff (including your Healthy School Coordinator, where possible) | What are the benefits and/or challenges of taking a team approach? How might you overcome some of these challenges? |
| 1. Meet as a team to develop an understanding of the Dashboard and to discuss your school data. | What topic areas are of relevance for your school and why? Come up with some actions to take forward over the next four weeks. |
| 1. Ahead of the next interview, reflect on how your team think this process has gone. | What worked well? What could be improved? |
